# Assessment of the sensitivity and specificity of serological (IFAT) and molecular (direct‐PCR) techniques for diagnosis of leishmaniasis in lagomorphs using a Bayesian approach

**DOI:** 10.1002/vms3.37

**Published:** 2016-06-15

**Authors:** María Luisa de la Cruz, Andres Pérez, Mercedes Domínguez, Inmaculada Moreno, Nerea García, Irene Martínez, Alejandro Navarro, Lucas Domínguez, Julio Álvarez

**Affiliations:** ^1^ Centro de Vigilancia Sanitaria Veterinaria VISAVET, Universidad Complutense Madrid Spain; ^2^ Department of Veterinary Population Medicine University of Minnesota St. Paul Minnesota USA; ^3^ Departamento de Inmunología Instituto de Salud Carlos III Majadahonda Madrid 28220 Spain

**Keywords:** *Leishmania infantum*, Lagomorphs, Diagnosis, IFAT, Ln‐PCR, Bayesian modelling

## Abstract

Leishmaniasis, caused by *Leishmania infantum*, is a vector‐borne zoonotic disease that is endemic to the Mediterranean basin. The potential of rabbits and hares to serve as competent reservoirs for the disease has recently been demonstrated, although assessment of the importance of their role on disease dynamics is hampered by the absence of quantitative knowledge on the accuracy of diagnostic techniques in these species. A Bayesian latent‐class model was used here to estimate the sensitivity and specificity of the Immuno‐fluorescence antibody test (IFAT) in serum and a *Leishmania*‐nested PCR (Ln‐PCR) in skin for samples collected from 217 rabbits and 70 hares from two different populations in the region of Madrid, Spain. A two‐population model, assuming conditional independence between test results and incorporating prior information on the performance of the tests in other animal species obtained from the literature, was used. Two alternative cut‐off values were assumed for the interpretation of the IFAT results: 1/50 for conservative and 1/25 for sensitive interpretation. Results suggest that sensitivity and specificity of the IFAT were around 70–80%, whereas the Ln‐PCR was highly specific (96%) but had a limited sensitivity (28.9% applying the conservative interpretation and 21.3% with the sensitive one). Prevalence was higher in the rabbit population (50.5% and 72.6%, for the conservative and sensitive interpretation, respectively) than in hares (6.7% and 13.2%). Our results demonstrate that the IFAT may be a useful screening tool for diagnosis of leishmaniasis in rabbits and hares. These results will help to design and implement surveillance programmes in wild species, with the ultimate objective of early detecting and preventing incursions of the disease into domestic and human populations.

## Introduction

Leishmaniasis is a term that refers to a group of vector‐borne diseases caused by parasites of the genus *Leishmania*, affecting humans and other mammals (Banuls *et al*. 2007). Leishmaniasis is considered one of the most important neglected diseases and it remains endemic in at least 88 developing countries. In addition, it has recently gained attention due to its emergency in Central and Northern European countries (Gramiccia & Gradoni 2005; Ready 2010). In the Mediterranean area, where leishmaniasis is endemic, *L. infantum* is the agent responsible for the disease (Boelaert *et al*. 2000), and sandflies of the genus *Phlebotomus* (*P. perniciosus* and *P. ariasi*) are the main vectors (Aransay *et al*. 2003; Franco *et al*. 2010; Galvez *et al*. 2011; Maroli *et al*. 2013), whereas dogs are considered the most important domestic reservoir in the region (Galvez *et al*. 2010; Gallego 2004). Other mammals that may be infected under field conditions include black rats, horses (Solano‐Gallego *et al*. 2003), cats (Martin‐Sanchez *et al*. 2007), rabbits (Chitimia *et al*. 2011; Garcia *et al*. 2014; Moreno *et al*. 2014), hares (Molina *et al*. 2012; Moreno *et al*. 2014) and a number of wild carnivores (Criado‐Fornelio *et al*. 2000; Millan *et al*. 2011; Sobrino *et al*. 2008).

A recent leishmaniasis outbreak in which 560 people were affected in the south‐western area of the province of Madrid, Spain, (Arce *et al*. 2013; Jimenez *et al*. 2014; Gomez‐Barroso *et al*. 2015) demonstrated the potential role that rabbits and hares may have as competent reservoirs for the disease. *L. infantum* infection was evidenced in the population of lagomorphs in the area of the outbreak using PCR and indirect immunofluorescence antibody test (IFAT), with up to 74.1% and 45.7% of seropositive hares and rabbits, respectively (Moreno *et al*. 2014). Another study carried out on an area of Madrid different from the region where the outbreak occurred revealed that 82.6% of the rabbits were positive to IFAT and/or PCR (Garcia *et al*. 2014), thus suggesting that the infection is prevalent in those species in that region. This finding may be important on the epidemiological dynamics of the disease given that rabbits and hares represent a large proportion of the mammalian biomass in Spain (Diaz‐Saez *et al*. 2014).

There are a number of biological characteristics of lagomorphs that are consistent with their potential role as competent reservoirs for *L. infantum*. Rabbits and hares’ life‐cycle is longer than the parasite‐transmission cycle. Furthermore, they are known to be a highly attractive blood source for *P. perniciosus* (Benito‐De Martin *et al*. 1994; Jimenez *et al*. 2013). Absence of clinical signs in most, if not all, the infected population allow for the continuous exposure to the vector, thus allowing further transmission (Diaz‐Saez *et al*. 2014). In addition, warrens of those species are a suitable biotope for the vector. Finally, there are experimental evidences demonstrating the ability of infected rabbits and hares to transmit *Leishmania* to a competent vector (*P. perniciosus*) (Jimenez *et al*. 2014; Molina *et al*. 2012).

Prevalence of *L. infantum* in the population of lagomorphs in infected settings is difficult to estimate due to the large uncertainties that exist on the performance of diagnostic tests in these species. Serological tests have not been properly optimized and assessment of the performance of direct‐PCR detection‐based tests is impaired by the lack of knowledge regarding pathogenesis of the disease in lagomorphs. Accuracy of serodiagnosis may also be affected by the occurrence of serological cross reactions between this parasite and *Trypanosoma nabiasi* (Diaz‐Saez *et al*. 2014), which is also prevalent in Spain.

On the other hand, sensitivities of DNA‐based tests seem to be variable, depending on the choice of the target sequence, the sample analysed and the target population of the assay (i.e. detection of exposure vs. active infection) (Lachaud *et al*. 2002). Different samples can be used for molecular detection, but the sensitivity of the test will depend on the stage of infection (Paradies *et al*. 2010). For that reason, the criterion for selection of sampled individuals may lead to false negative results, impairing the test sensitivity. In addition, PCR may fail to amplify parasite DNA when it is present at a low load (Hitakarun *et al*. 2014).

To assess the performance of *Leishmania* diagnostic tests in lagomorphs, which is a prerequisite for the development of surveillance and control strategies, a latent‐class analysis was performed on the IFAT and direct‐PCR results obtained on 217 rabbit samples and 70 hare samples from two different populations in the region of Madrid, Spain. This information will help to design and implement prevention and control programmes for the disease in Spain, and other endemically infected regions worldwide.

## Materials and methods

### Study population

Sample size was estimated assuming an expected prevalence of 50%, an error of 6% and a 95% confidence level, and an infinite lagomorph population size (Thrusfield 2005). Samples were collected from two areas of the region of Madrid, in central Spain, referred to as Northwestern (NW) and Northeastern (NE) sampling areas. Because of differences in the population density and species distribution, sampling was subsequently stratified, so that 75% and 25% of the samples were collected from rabbits in the NW and hares in the NE areas, respectively. Final sample sizes were 200 (rabbits, NW) and 67 (hares, NE), respectively. Finally, a total of 217 European rabbits (*Oryctolagus cuniculus*) and 70 hares (*Lepus granatensis*) were captured using ferreting (Cowan 1984) and nets, respectively. Samples were obtained between September and November 2013. This experimental design was approved by the Health and Environment authorities of the Madrid Council.

Animals (rabbits and hares) were transported to the laboratory within the first 5 h following their capture, where they were anaesthetized with a blend of ketamine (15 mg/kg) and xylazine (2–3 mg/kg) administered by intramuscular injection to ease the cardiac blood sampling. T61 (0.5–1 mL/animal) was then employed for their euthanasia and animals were necropsied to observe macroscopic lesions compatible with subclinical infections. In addition, information on the gender (determined by observing the external genitalia) and age (established based on the presence of cartilage conjunction in the ulna of the forelegs (Ballesteros 1998) was collected in 192 (88.5%) of the 217 rabbits (55 male and 137 female, 72 young and 120 adult) and in 64 (91.4%) of the 70 hares (31 male and 33 female, 23 young and 41 adult).

Serum, skin (from the external ear) and spleen samples were collected from all animals for laboratory determinations. Sera were analysed using IFAT; the skin and the spleens were stored at −20°C for subsequent PCR analyses.

### IFAT analysis

Rabbit and hare serum titres against *L. infantum* were obtained following procedures described elsewhere (Moreno *et al*. 2014). Briefly, 24‐well glass slides coated with 2 × 10*E*5 *L. infantum* (MCAN/ES/97/10 445) zymodeme MON‐1 grown for 5 *in vitro* passages were used. Serum samples (10 *μ*L) were analysed by serial doubling dilution (1/25 to 1/100) in PBS and incubated for 30 min at 37°C. Slides were washed three times (10 min each) in PBS, and 10 *μ*L of fluorescein‐labelled goat anti‐rabbit immunoglobulin (4050‐02; Southern Biotech, Birmingham, AL, USA) diluted in PBS supplemented with Evans blue (diluted 1/10^4^) were added to wells and incubated (37°C, 30 min). After incubation, slides were washed three times in PBS, mounted, and examined in a fluorescence microscope (Zeiss Axioskop 40; 40 ×  magnification). To detect anti‐*Leishmania* antibodies, a threshold value was established at 1/25 dilution (at this dilution, background antitrypanosomatid reactivity due to natural antibodies was negligible) using sera from *Leishmania*‐seronegative naïve NZW rabbits (Moreno *et al*. 2014). *L. infantum* promastigotes derived from various culture passages was used as the species‐specific target antigen. For genus‐specific antigen controls, low‐passage *L. amazonensis* promastigotes were used in parallel.

Finally, results of the IFAT titres ≥1/50 and <1/25 were classified as positive and negative, respectively, as previously described (Moreno *et al*. 2014). A titre between 1/25 and 1/50 was assumed to be inconclusive and alternatively considered as negative (‘conservative interpretation’) or positive (‘sensitive interpretation’) in further analyses.

### PCR analysis

DNA extraction: Approximately 25 mg of skin and 10 mg of spleen were placed in 300 *μ*L of NET‐10 buffer. DNA extraction was performed using the QIAamp Blood and Tissue kit (QIAGEN, Hilden, Germany) according to the procedure recommended by the manufacturer. DNA was resuspended in 150 *μ*L of elution buffer and frozen at −80°C until use. *Leishmania*‐nested PCR (Ln‐PCR): A specific *Leishmania*‐nested PCR reaction aimed at the SSU‐rRNA region (Cruz *et al*. 2002) was performed in all the skin samples. Negative (sterile water) and positive controls [DNA obtained from *L. infantum* (MCAN/ES/97/10 445) zymodeme MON‐1 promastigotes] were used on each assay. Reactions were carried out in a C1000 Thermal Cycler BIORAD (Alcobendas, Madrid, Spain). PCR products were visualized in a 2% agarose (Ultrapure Agarose, Invitrogen) gel using DNA SYBR Safe gel stain (Invitrogen) and 5 *μ*L loading marker (BIOTOOLS 100 bp Ladder Marker).

### Statistical analysis

Proportions of positive samples to each technique and individual characteristics were compared using Pearson's Chi‐square test. Agreement between the qualitative results recorded for both diagnostic techniques was measured using the kappa statistic, carried out with the SPSS software V. 20 (IBM Inc., Chicago, IL, USA).

A Bayesian latent‐class model was used to estimate the sensitivity and specificity of the IFAT test and the Ln‐PCR of skin samples considering that samples belonged to two different populations and results from both tests were conditionally independent (Gardner *et al*. 2000). This latter assumption was based on the very different principles underlying each technique (detection of specific antibodies vs. direct detection of DNA from the parasite). Still, the possible existence of conditional dependence was explored in a sensitivity analysis (Materials and methods, Statistical analysis).

**Figure 1:**
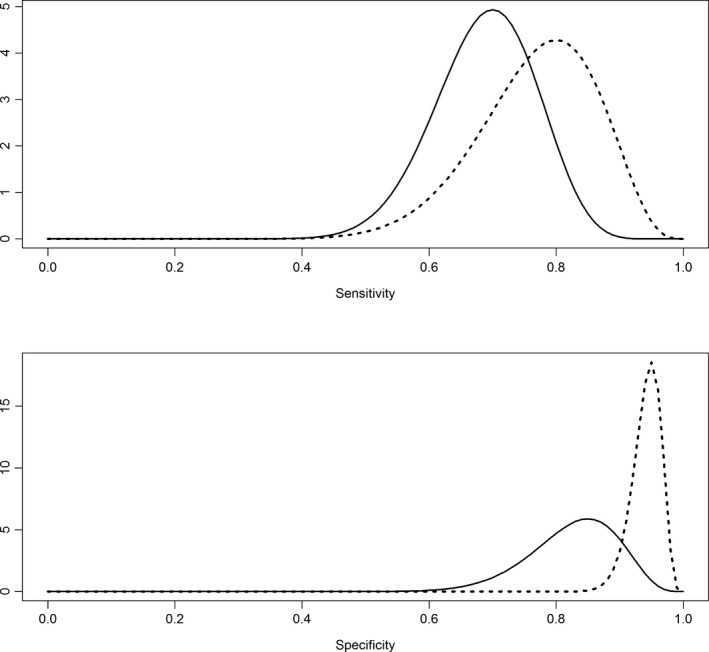
Beta distributions used as priors for the sensitivity and specificity of the immunofluorescence antibody test (IFAT) (solid line) and skin Ln‐PCR (dashed line) techniques.

Use of latent‐class models to estimate diagnostic tests accuracy in the absence of a gold standard has been described elsewhere (Branscum *et al*. 2005; Gardner *et al*. 2000). Briefly, the value of parameters (sensitivity and specificity of each technique, prevalence) is jointly estimated (posterior probability) considering the uncertainty about the true value of those parameters (prior distributions) and the collected data. Given the lack of data about the performance of the tests in lagomorphs, evidences obtained in dogs and, to a lesser extent, in humans and cats, were used. Prior distributions for the sensitivity and specificity of the diagnostic tests were fitted using information obtained from the literature (Table S1) and expert opinion of two of the authors (D.M., M.I.). Sensitivity and specificity were assumed to be beta‐distributed (Fig. 1, Table 2). Given the lack of information about the possible prevalence in the studied populations, we used a beta prior distribution (assuming a most likely value of 40% and 95% confidence it was below 80%) in agreement with results previously estimated for lagomorphs in a different area of the Madrid region (Moreno *et al*. 2014).

A sensitivity analysis was conducted to elucidate if the model was robust to the selection of priors. For the sensitivity analysis, non‐informative 0–1 uniform distributions were used alternatively as the prior for the sensitivity and specificity of each test and the expected prevalence. Model estimates obtained using the non‐informative distribution for all five parameters were then compared with those obtained using the informative priors, and overlapping of 95% Bayesian posterior probability intervals (PPI) was considered indicative of model robustness. A model including the results of the Ln‐PCR of both skin and spleen samples (parallel interpretation) was also run to try to improve the sensitivity of the test. Finally, an alternative model considering that tests were conditionally dependent was also evaluated.

All analyses were implemented in WinBugs 1.4.3 (Lunn *et al*. 2000). Three Markov chain Monte Carlo runs were performed to visually assess convergence and mixing. Convergence was also assessed by evaluating that the Gelman–Rubin value was below 1.1 (Gelman & Rubin 1992). The mode of the prior distributions was used as the initial value for each estimated parameter in each chain and models were run for 14 500 iterations after discarding the first 500 burn‐in for computing the posterior estimates, and autocorrelation was eliminated by thinning the samples by collecting one in 10 consecutive samples.

## Results

### Descriptive results

Two rabbits and one hare could not be sampled for PCR testing, and were therefore excluded from the analysis. From the remaining 284 animals, 215 were rabbits and 69 were hares. A total of 107 rabbits (49.8%) tested IFAT‐positive, whereas 73 (34%) and 35 (16.3%) were negative and inconclusive, respectively. Proportion of reactors in the IFAT in the rabbit population therefore ranged from 49.8% to 66% when the conservative/sensitive interpretation was in place (Table 1 and Table S2). In contrast, only 26 rabbits (12.1%) tested positive in the Ln‐PCR of skin samples. This number increased to 28 if results from both skin and spleen samples were considered in parallel.

**Table 1:** Number of reactors to the IFAT test and to the Ln‐PCR (skin samples) performed on 215 rabbits and 69 hares in Madrid (Spain) for the combination of diagnostic tests and IFAT interpretation criteria

Species	Interpretation IFAT test	IFAT+/PCR+	IFAT+/PCR‐	IFAT‐/PCR+	IFAT‐/PCR‐
Rabbits	Conservative	16	91	10	98
	Sensitive	19	123	7	66
Hares	Conservative	0	12	1	56
	Sensitive	0	21	1	47

IFAT, immunofluorescence antibody test

The number of reactors was significantly (*P* < 0.001) lower in the hare population, with 12 (17.4%), 48 (69.6%) and 9 (13%) samples being positive, negative and inconclusive to the IFAT test, respectively, whereas only one skin sample tested positive to the Ln‐PCR (Table 1 and Table S2).

No gross pathology compatible with disease was observed in any case. A significantly (*P* < 0.001) higher proportion of seropositive adults was observed (74/159, 46.5%) compared with young animals (26/94, 27.7%). When considering Ln‐PCR results, no significant differences depending on age and gender of the animals were detected.

Tests agreement was very low regardless the interpretation used in the IFAT test (Kappa = 0.076 using a conservative interpretation and 0.044 with the sensitive interpretation).

### Model results

Using the two‐population model, posterior estimates of the sensitivity of the IFAT test were in general higher than the prior used in the model, whereas the opposite was true for the specificity, but in both cases there was a large overlap between prior and posterior values, with both median posterior estimates >70% (Table 2). This was not the case for the Ln‐PCR, because the median posterior estimates for the sensitivity were below 30% regardless which interpretation was used for the IFAT. Large uncertainties associated with those estimates resulted in large 95% PPI, particularly when a conservative interpretation of the IFAT was applied (Table 2). In contrast, high estimates were obtained for the specificity of this technique.

**Table 2:** Prior (mode and low/up bound for 95% cumulative probability) and posterior estimates (median and 95% Bayesian posterior probability interval) for sensitivity, specificity and prevalence of infection (%) obtained for the combination of diagnostic test and IFAT interpretation criteria on 284 lagomorphs from Madrid (Spain) using a two‐population model

	IFAT test estimates		Nested‐PCR (skin samples) estimates		Prevalence	
	Sensitivity	Specificity	Sensitivity	Specificity	P rabbits	P hares
Prior estimates	70 (95% cumulative probability>55)	85 (95% cumulative probability>70)	80 (95% cumulative probability>60)	95 (95% cumulative probability>90)	40 (95% cumulative probability<80)	
Beta distributions						
A	22.5135	23.9027	14.8442	99.6983	2.0591	
B	10.2201	5.0417	4.4611	6.1946	2.5886	

Posterior estimates						
Diagnostic interpretation						
IFAT conservative int.	73 (61.3–83.8)	80.2 (62.9–90.8)	28.9 (19.2–70.8)	96.4 (93.3–98.4)	50.5 (13.9–70.1)	6.7 (0.9–22)
IFAT sensitive int.	78.2 (68.2–86.8)	75.9 (61.7–88.8)	21.3 (14.8–31.2)	96.2 (92.7–98.3)	72.6 (51–89.5)	13.2 (1.9–34.7)

The estimated prevalence was higher in the rabbit population, compared with hares (Table 2) as a result of the higher number of reactors detected using both techniques (Table 1). The model converged properly as indicated by the visual inspection of the chains and the Gelman–Rubin statistic <1.1 for all estimated parameters (Additional details are available in Table S3).

No major changes in the posterior estimates were observed when a non‐informative prior was used regardless the model used (Table S4).

Inclusion of spleen results did not changed the sensitivity of the Ln‐PCR, yielding values of 29.6% (95% probability interval: 19.7–68.1) and 21.8% (95% probability interval: 15.4–31.3) for the conservative and sensitive interpretation, respectively.

Assumption of conditional independence between the IFAT and the Ln‐PCR test results was supported by the results of the model including a conditional dependence term, with a 95% PPI that encompassed 0.

## Discussion

Due to the lack of an acceptable gold‐standard (Marfurt *et al*. 2003), a Bayesian analysis was performed here to estimate the field diagnostic sensitivity and specificity of two diagnostic techniques commonly used for detection of infection by *L. infantum* in lagomorphs. This is the first time that this approach is used to evaluate the performance of diagnostic tests in the case of leishmaniasis. Our results demonstrate that the IFAT may be a useful screening tool at the population level, although has a limited specificity. The Ln‐PCR using ear skin, on the other hand, lacked sensitivity in the populations of lagomorphs analysed.

Posterior estimates for the sensitivity of the IFAT using the conservative interpretation were in agreement with the prior information, extracted mainly from publications on dogs, humans and, to a lesser extent, cats, whereas the use of a sensitive criterion resulted in an increase of around 5–10%. These results, coupled with evidence suggesting that the titre of antibodies in lagomorphs is generally higher than that observed in humans or dogs (Pastoret *et al*., 1998), further supports the potential use of this technique as a fast and affordable screening tool. In addition, the ability of the technique to detect both IgM and IgG may allow the detection of infection in early stages. In this study promastigotes obtained after no more than six culture passages were used to obtain the antigen for the IFAT, what could also contribute to an enhanced sensitivity as previously demonstrated in lagomorphs (Moreno *et al*. 2014).

Specificity of the IFAT was also in the range of the prior information used in the model, although values were lower when a sensitive interpretation was used (Table 2). This result may be explained, at least in part, by the occurrence of false positive reactions caused by cross‐reactivity to Trypanosome spp (Moreno *et al*. 2014), whose presence has been demonstrated, at least, in southern Spain (Diaz‐Saez *et al*. 2014).

In contrast, performance of the Ln‐PCR was particularly limited in terms of sensitivity, with median posterior estimates between 20 and 30%, what could be related with a low parasitic load or a limited analytic sensitivity of the PCR. A relatively high parasite load in the skin would be expected in the early stages of infection, as demonstrated in experimental infections in mice (Belkaid *et al*. 2000; Kamhawi *et al*. 2000; Nicolas *et al*. 2000) and asymptomatic dogs (Otranto *et al*. 2009; Solano‐Gallego *et al*. 2001), in which *Leishmania* DNA concentration was higher after a recent inoculation of the promastigotes by the phlebotomus. However, rabbits and hares analysed in this study were captured at the end of the transmission season and therefore could have been exposed early in the year (or in previous seasons). This result is compatible with a large proportion of seropositive animals, as found in both populations, given the duration of the immunity. The lack of detection of macroscopical changes typical of clinical disease in the analysis post‐mortem also suggests that infection may have not been severe. In addition, the occurrence of a ‘parasite silencing stage’ in which the parasite load drops to undetectable levels for a variable period of time until the number of organism increases again, leading to Ln‐PCR false negative results, as already described in natural and experimental *L. infantum* infection in dogs (Oliva *et al*. 2006; Paranhos‐Silva *et al*. 2003), cannot be ruled out.

Another potential explanation for the limited sensitivity of the Ln‐PCR would be a lack of analytic sensitivity, i.e. the inability to detect parasitic DNA in the sample. Still, previous studies have reported a high analytic sensitivity of this PCR, being able to detect DNA from as low as 0.01 promastigote from a *Leishmania* culture (Cruz *et al*. 2002).

The lack of agreement between IFAT and Ln‐PCR observed in our study is likely the result of the different subpopulations targeted by each technique, with IFAT detecting both present but especially past infections regardless parasitic load (Berrahal *et al*. 1996; Mary *et al*., 1999) and Ln‐PCR being most sensitive in the case of acute infections (Diaz‐Saez *et al*. 2014). This result is consistent with the assumption of independence between tests results initially assumed here, which was confirmed in the sensitivity analysis conducted to test such assumption.

Relatively informative priors were used in the analysis based on previous knowledge. The effect of this selection was explored through the sensitivity analysis, in which results of the two‐population model were considered reliable due to the high stability of estimates when their priors were replaced by non‐informative distributions.

The significantly higher prevalence estimated in rabbits compared with hares may be due to a different susceptibility to infection. However, the competency of both species to act as reservoirs for *Leishmania* has been demonstrated (Jimenez *et al*. 2014; Molina *et al*. 2012), and therefore differences may be also due to a different infectious pressure on each population. This hypothesis is consistent with the observation that both areas of study are separated by more than 25 km, and highlights the variability that may be found under field conditions in terms of prevalence due to other environmental factors not related with the host species (such as vector density). In addition, frequent aggregation of rabbits in warrens could also favour disease transmission if the vector was present, as this species is known to be a highly attractive blood source for *P. perniciosus* (Benito‐De Martin *et al*. 1994; Jimenez *et al*. 2014; Martin‐Martin *et al*. 2014), what could also explain at least partly differences with the results in hares.

In conclusion, results here provide quantitative estimates of the accuracy of diagnostic tests for detection of leishmaniosis in lagomorphs. These results will help to design effective strategies for prevention of disease introduction into susceptible animal and human populations in endemically infected areas.

## Source of funding

This work is a contribution to the EU FP7 ANTIGONE Project (278976) and was partially supported by structural funds of the European Union, S2013/ABI‐2747 (TAVS), and by the program I+D “Vigilancia Sanitaria” from the local government of Madrid.

## Conflicts of interest

The authors declare that they have no con?icts of interest.

## Contributions

MLdlC, and AP performed the statistical analyses and drafted the manuscript. NG designed the sample collection and supervised the laboratory testing. MD, IM1, IM2 and AN collected the samples and performed laboratory analyses. LD and JA designed the study, coordinated the work and participated in the data analysis and interpretation.

## Supporting information


**Table S1**. IFAT and skin Ln‐PCR Sensitivity and specificity reported values in the literature.
**Table S2**. Distribution of antibody titres of 215 rabbits and 69 hares included in the study.
**Table S3**. MC errors and Gelman–Rubin statistic of posterior estimates obtained for the combination of diagnostic test and IFAT interpretation criteria in a two‐population model.
**Table S4**. Posterior estimates (median and 95% Bayesian posterior probability interval) for sensitivity, specificity and prevalence of infection (%) obtained for the combination of diagnostic test and IFAT interpretation criteria on 284 lagomorphs from Madrid (Spain) when non‐informative priors were used in the two‐population model.
