# Supplementary material for: Assessment of the sensitivity and specificity of serological (IFAT) and molecular (direct‐PCR) techniques for diagnosis of leishmaniasis in lagomorphs using a Bayesian approach
Source: Vet Med Sci. 2016 Jun 15;2(3):211–20. doi: 10.1002/vms3.37 (PMC5645865; doi:10.1002/vms3.37)
Supplement: Supplementary file 1 — Table S1. IFAT and skin Ln‐PCR Sensitivity and specificity reported values in the literature. Table S2. Distribution of antibody titres of 215 rabbits and 69 hares included in the study. Table S3. MC errors and Gelman–Rubin statistic of posterior estimates obtained for the combination of diagnostic test and IFAT interpretation criteria in a two‐population model. Table S4. Posterior estimates (median and 95% Bayesian posterior probability interval) for sensitivity, specificity and prevalence of infection (%) obtained for the combination of diagnostic test and IFAT interpretation criteria on 284 lagomorphs from Madrid (Spain) when non‐informative priors were used in the two‐population model. [file VMS3-2-211-s001.doc]

Supplementary Table 1

IFAT and skin Ln-PCR Sensitivity and specificity reported values in the literature.

| **Diagnostic test** | **Reference** | **Species/sample** | **Sensitivity (%)** | **Specificity (%)** |
| --- | --- | --- | --- | --- |
| **IFAT** | Chatzis et al., 2014 | Cat/serum | -- | 100 |
|  | Iqbal et al., 2002 | Human/serum | 86.6 | 93.0 |
|  | Manna et al., 2004 | Dog/serum | 85.3 | -- |
|  | Marfurt et al., 2003 | Human/serum | 57.1 | 100 |
|  | Mettler et al., 2005 | Dog/serum | 90 | 100 |
|  | Silva et al., 2001 | Dog/serum | 21.6 | -- |
|  | Bernadina et al., 1997 | Dog/serum | 100 | -- |
|  | Maia et al., 2007 | Dog/serum | 85.5 | 94.7 |
|  | Ashford et al., 1995 | Dog/serum | 63 | -- |
|  | Solano-Gallego et al., 2014 | Dog/serum | 86.9 | 91.7 |
| **LnPCR**  **(skin sample)** | Gomes et al., 2007 | Dog/skin | 90.7 | 93.3 |
|  | Manna et al., 2004 | Dog/skin | 95 | -- |
|  | Marfurt et al., 2003 | Human/skin | 51.7 | 100 |
|  | Ashford et al., 1995 | Dog/skin | 100 | 60-100 |
|  | Mohammadiha et al., 2013 | Dog/serum | 67.6 (asympt.) | 100 |
|  | Carvalho Ferreira et al., 2014 | Dog/skin | 63.3 (asympt.) | 100 |

Supplementary Table 2

Distribution of antibody titers of lagomorphs included in the study

| Species | **IFAT antibody titers (n animals)** | | | | **Total** |
| --- | --- | --- | --- | --- | --- |
|  | **<1/25** | **≥1/25 and <1/50** | **≥1/50 and ≤1/100** | **> 1/100** |  |
| Rabbits | 73 (34%) | 35 (16.3%) | 74 (34.4%) | 33 (15.3%) | 215 |
| Hares | 48 (69.6%) | 9 (13%) | 11 (15.9%) | 1 (1.5%) | 69 |

Supplementary Table 3

MC errors and Gelman-Rubin statistic of posterior estimates obtained for the combination of diagnostic test and IFAT interpretation criteria in a two population model.

|  | IFAT conservative interpretation | | IFAT sensitive interpretation | |
| --- | --- | --- | --- | --- |
|  | **MC error** | **Gelman-Rubin statistic** | **MC error** | **Gelman-Rubin statistic** |
| IFAT sensitivity | 9.15E-4 | 1.0009 | 6.95E-4 | 1.0010 |
| IFAT specificity | 1.49E-3 | 1.0057 | 1.12E-3 | 1.0013 |
| Ln-PCR (skin) sensitivity | 2.75E-3 | 1.0025 | 7.91E-4 | 1.0016 |
| Ln-PCR (skin) specificity | 2.01E-4 | 1.0022 | 2.16E-4 | 1.0008 |
| P rabbits | 2.91E-3 | 1.0050 | 1.64E-3 | 1.0123 |
| P hares | 8.22E-4 | 1.0007 | 1.39E-3 | 1.0007 |

Supplementary Table 4

Posterior estimates (median and 95% Bayesian posterior probability interval) for sensitivity, specificity and prevalence of infection (%) obtained for the combination of diagnostic test and IFAT interpretation criteria on 284 lagomorphs from Madrid (Spain) when non-informative priors were used in the two population model.

|  | **Non-informative prior** | | **Posterior estimates** | | | | | | | | | | |
| --- | --- | --- | --- | --- | --- | --- | --- | --- | --- | --- | --- | --- | --- |
|  |  |  | IFAT test estimates | | | | Nested-PCR (skin samples) estimates | | | | P rabbits | | P hares |
|  |  |  | Sensitivity | | Specificity | | Sensitivity | | Specificity | |  |  |  |
| **IFAT conservative interpretation** | IFAT sensitivity | 78.6 (59.5-96.5) | | 80.1 (63-90.9) | | 29.8 (19.4-69.4) | | 96 (92.7-98.3) | | 46.1 (13.8-68.7) | | 6.2 (0.93-20) | |
|  | IFAT specificity | 71.5 (57.6-83.1) | | 65.9 (56.3-89.9) | | 49.5 (21-85.5) | | 96.4 (93.3-98.5) | | 24.1 (8.61-65.6) | | 4.6 (0.67-17.8) | |
|  | PCR sensitivity | 69.1 (56.2-81.6) | | 85.9 (76.3-93.9) | | 15.9 (9.83-24.2) | | 95.8 (92.2-98.2) | | 63.8 (46.7-83.3) | | 11.5 (1.99-28.9) | |
|  | PCR specificity | 71.8 (59.9-82.6) | | 79.7 (62.5-90.5) | | 29.9 (19.6-71.6) | | 98.3 (94-99.9) | | 50.8 (15.7-70.7) | | 6.86 (1.04-21.3) | |
|  | P rabbits | 72.7 (60.2-83.7) | | 80.3 (62-91.1) | | 28.7 (18.7-74.8) | | 96.4 (93.2-98.4) | | 51 (12.1-72.7) | | 6.66 (0.94-21.5) | |
|  | P hares | 73.4 (60.7-84.1) | | 77.7 (61.5-88.9) | | 31.3 (20-76.8) | | 96.4 (93.3-98.4) | | 46.3 (12-67.7) | | 2.7 (0.1-15) | |
|  | Results final model (using informative priors) | **73**  **(61.3-83.8)** | | **80.2**  **(62.9-90.8)** | | **28.9**  **(19.2-70.8)** | | | **96.4**  **(93.3-98.4)** | | **50.5**  **(13.9-70.1)** | | **6.7**  **(0.9-22)** |
| **IFAT sensitive interpretation** | IFAT sensitivity | 87.2 (72.5-98.7) | | 75.8 (62-88.9) | | 22.6 (15.6-32.9) | | 95.8 (92.3-98.2) | | 64.1 (44.3-83) | | 11.7 (1.76-31.5) | |
|  | IFAT specificity | 74.4 (60.1-85.4) | | 46.8 (39.2-77.1) | | 61.3 (18.5-87.4) | | 96.5 (93.4-98.6) | | 18.2 (7.85-78.4) | | 4.55 (0.68-20) | |
|  | PCR sensitivity | 75.3 (65.9-85) | | 80.5 (67.9-91.9) | | 13.2 (8.52-19.4) | | 95.8 (91.9-98.2) | | 80.6 (64.6-94.1) | | 20.6 (4.11-42.3) | |
|  | PCR specificity | 77.6 (68.2-86.2) | | 75.5 (61.1-88.4) | | 21.7 (15.1-32) | | 98.2 (93.2-99.9) | | 73 (51.3-88.9) | | 13.2 (2.05-34.7) | |
|  | P rabbits | 76.3 (65.9-86.1) | | 77.5 (63.9-90.1) | | 20.1 (14.1-28.8) | | 96.2 (92.7-98.4) | | 77.5 (56.6-96.1) | | 15 (2.34-37.9) | |
|  | P hares | 78.4 (68.6-87.2) | | 73.3 (57.9-86.4) | | 22.1 (15.3-34.9) | | 96.3 (92.9-98.4) | | 70.4 (43.4-87.3) | | 6.43 (0.23-29.6) | |
|  | Results final model (using informative priors) | **78.2**  **(68.2-86.8)** | | **75.9**  **(61.7-88.8)** | | **21.3**  **(14.8-31.2)** | | | **96.2**  **(92.7-98.3)** | | **72.6**  **(51-89.5)** | | **13.2**  **(1.9-34.7)** |

References

Ashford, D.A., M. Bozza, M. Freire, J.C. Miranda, I. Sherlock, C. Eulalio, U. Lopes, O. Fernandes, W. Degrave, R.H. Jr. Barker et al., 1995: Comparison of the polymerase chain reaction and serology for the detection of canine visceral leishmaniasis. The American journal of tropical medicine and hygiene 53, 251-255.

Bernadina, W.E., R. De Luna, G. Oliva, P. Ciaramella, 1997: An immunodiffusion assay for the detection of canine leishmaniasis due to infection with *Leishmania infantum*. Veterinary parasitology 73, 207-213.

Carvalho Ferreira, A.L., V.M. Carregal, S. de Almeida Ferreira, R.S. Leite, A.S. de Andrade, 2014: Detection of *Leishmania infantum* in 4 different dog samples by real-time PCR and ITS-1 nested PCR. Diagnostic microbiology and infectious disease 78, 418-421.

Chatzis, M.K., L. Leontides, L.V. Athanasiou, E. Papadopoulos, D. Kasabalis, M. Mylonakis, T. Rallis, A.F. Koutinas, M. Andreadou, J. Ikonomopoulos, M.N. Saridomichelakis, 2014: Evaluation of indirect immunofluorescence antibody test and enzyme-linked immunosorbent assay for the diagnosis of infection by *Leishmania infantum* in clinically normal and sick cats. Experimental parasitology 147, 54-59.

Gomes, A.H., I.M. Ferreira, M.L. Lima, E.A. Cunha, A.S. Garcia, M.F. Araujo, V.L. Pereira-Chioccola, 2007: PCR identification of *Leishmania* in diagnosis and control of canine Leishmaniasis. Veterinary parasitology 144, 234-241.

Iqbal, J., P.R. Hira, G. Saroj, R. Philip, F. Al-Ali, P.J. Madda, A. Sher, 2002: Imported visceral leishmaniasis: diagnostic dilemmas and comparative analysis of three assays. Journal of clinical microbiology 40, 475-479.

Maia, C., N. Rolao, M. Nunes, L. Goncalves, L. Campino, 2007: Infectivity of five different types of macrophages by *Leishmania infantum*. Acta tropica 103, 150-155.

Manna, L., F. Vitale, S. Reale, S. Caracappa, L.M. Pavone, R.D. Morte, G. Cringoli, N. Staiano, A.E. Gravino, 2004: Comparison of different tissue sampling for PCR-based diagnosis and follow-up of canine visceral leishmaniosis. Veterinary parasitology 125, 251-262.

Marfurt, J., A. Nasereddin, I. Niederwieser, C.L. Jaffe, H.P. Beck, I. Felger, 2003: Identification and differentiation of *Leishmania* species in clinical samples by PCR amplification of the miniexon sequence and subsequent restriction fragment length polymorphism analysis. Journal of clinical microbiology 41, 3147-3153.

Mettler, M., F. Grimm, G. Capelli, H. Camp, P. Deplazes, 2005: Evaluation of enzyme-linked immunosorbent assays, an immunofluorescent-antibody test, and two rapid tests (immunochromatographic-dipstick and gel tests) for serological diagnosis of symptomatic and asymptomatic *Leishmania* infections in dogs. Journal of clinical microbiology 43, 5515-5519.

Mohammadiha, A., A. Haghighi, M. Mohebali, R. Mahdian, A.R. Abadi, Z. Zarei, F. Yeganeh, B. Kazemi, N. Taghipour, B. Akhoundi, M. Barati, M.R. Mahmoudi, 2013: Canine visceral leishmaniasis: a comparative study of real-time PCR, conventional PCR, and direct agglutination on sera for the detection of *Leishmania infantum* infection. Veterinary parasitology 192, 83-90.

Silva, E.S., C.M. Gontijo, R.S. Pacheco, V.O. Fiuza, R.P. Brazil, 2001: Visceral leishmaniasis in the Metropolitan Region of Belo Horizonte, State of Minas Gerais, Brazil. Memorias do Instituto Oswaldo Cruz 96, 285-291.

Solano-Gallego, L., S. Villanueva-Saz, M. Carbonell, M. Trotta, T. Furlanello, A. Natale, 2014: Serological diagnosis of canine leishmaniosis: comparison of three commercial ELISA tests (Leiscan, ID Screen and *Leishmania* 96), a rapid test (Speed Leish K) and an in-house IFAT. Parasites & vectors 7, 111.
